# PTSD in French Adolescent Victims Following the London Attack in March 2017: Data From the First Step of the AVAL Study

**DOI:** 10.3389/fpsyt.2022.728133

**Published:** 2022-02-24

**Authors:** Nathalie Coulon, Marion Grenon, Maëlys Consigny, J-P Simson

**Affiliations:** ^1^Department of Psychiatry, Brest University Hospital, Brest, France; ^2^Assistance Publique des Hôpitaux de Paris APHP, DHU Pe-PSY, Medical University Department of Psychiatry and Addictology Henri Mondor, Schizophrenia Expert Center, Paris Est Créteil University, Créteil, France; ^3^Military Hospital, HIA Clermont-Tonnerre, Departement of Psychiatry, Brest, France; ^4^Brest University Hospital, Delegation for Clinical Research and Innovation, Clinical Investigation Center, INSERM 14 12, Brest, France

**Keywords:** terrorism, Post-Traumatic Stress Disorder, adolescent psychiatry, teenagers (adolescence), trauma

## Abstract

**Background:**

The terrorist attack at Westminster Bridge on March 22^nd^, 2017 impacted on French high school students on a school trip in London. This terrorist attack was claimed by the Islamic State. The aim of the study was to assess the mental health consequences of the attack on the French adolescents who were directly exposed (criteria A for *Post-Traumatic Stress Disorder*, PTSD). This involved three dimensions, namely: (1) clinical; (2) epidemiological; and (3) prevention and therapeutic.

**Material and Method:**

The investigation was the first observational step of AVAL (*Adolescents Victimes de l'Attentat de Londres*) study, a cohort monitoring project and it was then a monocentric, cross sectional, non interventional survey, at only one-year post-trauma. The study was carried out utilizing self- and clinician-administered questionnaires. Volunteers from the medico-psychological emergency unit provided support for these victims during the study protocol.

**Results:**

From the target population (*n* = 53), 39 adolescents (73.6%) agreed to participate, with a median age 16.9 years. 12 months after the attack, 25.6% of teenagers suffered from current PTSD (*p* < 0.0001). Those with, vs. without, PTSD showed several significant differences: (1) heightened levels of major depressive episodes (*p* = 0.0266) and suicidality (*p* = 0.0164); (2) increased substance use, including tobacco (*p* = 0.0284) and cannabis (*p* = 0.0449); and (3) impaired functioning in school (*p* = 0.0203), social (*p* < 0.0001) and family (*p* < 0.0001) settings. Sixty four percentage of directly exposed teenagers also had a current psychiatric disorder other than PTSD.

**Discussion:**

The heightened levels of PTSD, psychiatric disorders, and substance use at 12 months highlight the importance of early intervention in adolescents exposed to terrorist-linked potentially traumatic events.

## Background

On March 22^nd^, 2017, a terrorist attack occurred in the district of Westminster in London, United Kingdom. The Islamic State claimed responsibility for this attack, which was the first in a series of five attacks in the UK in 2017, four of which occurred in London.

The March 2017 terrorist attack resulted in 6 fatalities (including the attacker), with 50 being injured, including 3 French high school students who were part of a larger party on a school trip. These French teenagers were present on Westminster Bridge, when the terrorist's car sped along the bridge and pavement, hitting pedestrians indiscriminately. These adolescents constitute the target population of this study. All of these teenagers were repatriated to France the following day, except for the three injured ones. The medico-psychological emergency unit was deployed to their school in order to provide psychosocial support for the children and their families. This attack had a large media coverage in the UK, but the French victim's high school protected them from media exposure.

Exposure to a traumatic event in childhood or adolescence can have a variety of consequences, ranging from relatively mild to serious, including long-term repercussions (1). Age seems a relevant modulator of the psychological consequences of trauma exposure, with regressive behaviors more prevalent in children aged 5 years and under, whilst school and attention disorders being more evident in children aged 6–11 years. Adolescents, aged 12–18 years, seem to have similar responses to adults (2, 3). A systematic review (4) of epidemiological studies among child and adolescent survivors of disasters between 1987–2011, showed a Post-Traumatic Stress Disorder (PTSD) rate, according to DSM IV (5), ranging from 1–60%, with depression being the second most commonly observed psychiatric diagnosis. Much of the variance in the emergence of PTSD arises from diverse nature of the experienced traumas, which included earthquakes, tsunamis, hurricanes and floods, as well as attacks. The importance of the nature of the trauma is highlighted by data in trauma-experiencing adults (6), which emphasizes the importance of intentional vs. non-intentional, usually human-induced vs. natural. This review showed the median prevalence of PTSD in the intentional trauma category to increase from 11.8% at 1-month post-trauma to 23.3% at 12 months, in contrast to the decrease over time following a natural trauma. A WHO systematic review and meta-analysis of 96 studies (7), with a robust methodology, estimated the age-standardized prevalence for PTSD to be elevated in conflict-affected populations (15.3% [9.9–23.5]).

As well as variance arising from age and disaster origin, the psychological disorders arising from trauma can be diverse (2, 3, 8, 9), including depression, anxiety, and somatic disorders, as well as substance abuse (10–12). Several risk and protective factors of PTSD, including pre-, peri- and post-traumatic factors, have also been identified. A meta-analysis (13) focusing on children and adolescents, aged 6–18 years, across 64 studies, revealed “*subjective peri-trauma factors*” (peri-traumatic distress and dissociation, perceived life threat) and “*post event factors*” (low social support, comorbid psychological problem, poor family functioning) to be relevant modulators of PTSD development.

This study aimed to investigate the mental health impact at one-year post-trauma arising from the terrorist attack that occurred in London on March 22^nd^, 2017 in a group of directly exposed French teenagers. The study looked at a number of factors, including: (1) clinical, namely the prevalence of PTSD and other psychological/psychiatric disorders, as well as changes in social, family and school functioning; (2) epidemiological, whereby risk factors were identified for the development of PTSD or other disorders and define the adolescents who received care, one year after the event; (3) preventive and therapeutic implications.

## Materials and Methods

### Design of the Study

The investigation was the first observational step of AVAL (*Adolescents Victimes de l'Attentat de Londres*) study, a cohort monitoring project. So, it was a first description of a series of investigations of a follow up of 53 French school teenagers, directly exposed to the terrorist attack in London on March 22^nd^, 2017 and then a prospective, monocentric, cross-sectional, non-interventional study, supported by a health cooperation group “*Groupement de Cooperation Sanitaire HospiBrest*”, which included the civilian university hospital and the military hospital of the city of Brest in France. The study took place ~1 year after the traumatic event, with recruitment occurring between March 14^th^, 2018 and April 13^th^, 2018. The design of the AVAL study has been detailed previously (14) and the evaluation consisted of both self-assessment questionnaires and clinician administered questionnaires, with research of exhaustiveness in the recruitment of participants, by going to students in their school. The security of the assessment was sought with the continuous presence of volunteers from the departmental medico-psychological emergency unit and only a single investigator for the clinician -administered questionnaires, with the supervision of two senior doctors.

### Inclusion Criteria

All study participants were teenagers, schooled in a French high school (near the city of Brest), and were directly exposed to the terrorist attack on Westminster Bridge in London, on March 22^nd^, 2017. Their direct exposure satisfied criterion A for PTSD in DSM 5 (15).

### Exclusion Criteria

School teenagers with no direct sensory exposure to the trauma were excluded, including the three high school students physically injured and high school students absent from the place of the attack. Teachers, accompanying adults and family/friends were also excluded. Preventive information, about trauma and therapy options, were given to all those directly or indirectly linked to the event but satisfying exclusion criteria.

### Clinical Data

The clinical evaluation was divided into two parts, each ~1 h long, comprising of self-administered questionnaires and clinician-administered questionnaires. The difficulty of event recall during such data collection was eased by the constant presence of volunteers from the departmental medico-psychological emergency unit.

#### Self-Administered Questionnaires

The self-administered questionnaires were used to collect socio-demographic information, risk and protective factors, as well as self-perceived consequences on school, social and family functioning and attendance at the trauma clinic. The self-questionnaire booklet therefore included (13): (1) socio demographics questions (age, sex, grade in school, social-economic level of parents or legal guardians, ethnic origin), (2) Life Event Checklist for DSM-5(LEC-5) (16), a list of difficult life events prior to the attack and (3) Adolescents Life-Change Events (ALCES) (17), in order to explore the potential risk factors for PTSD; (4) Sheehan Disability Scale (SDC) (18, 19), to measure the impact of psychiatric conditions on daily life, (5) Social Provision Scale-Short form (20), to assess the declared and perceived quality of social support received by the subject, (6) School and family resources close-ended questions and (7) School performances close-ended questions, to assess aspects of wider functioning; (8) Post-traumatic stress disorder Check-List for DSM-5 (PCL-5) (21) and Peritraumatic Distress Inventory(PDI) (22) to identify PTSD symptoms and peritraumatic emotional distress reactions.

The PCL is long-established questionnaire (23), used as a screening and diagnostic tool, that measures PTSD severity as well as prevalence in some studies (24). PCL has recently been revised and adapted to satisfy DSM-5 criteria, with high reliability and validity between PCL and PCL-5 (25). Consequently, PCL-5 was utilized in the current investigation, including the recommended PTSD diagnostic cut-off (21, 24). This allowed the division of the study participants into two groups (with or without PTSD), thereby allowing an evaluation of PTSD prevalence in this study population.

#### Clinician-Administered Questionnaires

Clinical and epidemiological data collected by the self-administered questionnaires were supplemented by: (1) The creation of a genogram over 3 generations to explore the history of personal and family mental illness; (2) The Mini International Neuro-psychiatric Interview for children and adolescents *(*M.I.N.I Kid 7.0.2), to investigate a broad range of psychiatric disorders (26); (3) Closed-ended question pertaining to the use of psychoactive substances, especially cannabis, tobacco, and alcohol use; (4) World Health Organization Disability Assessment Schedule 2.0 (WHODAS 2.0) to assess functional impairment (5); (5) Details of psychological and medical care following the attack to explore the healthcare pathways followed by the participants, including the type of care received.

### Ethical Concerns

The study was carried out in accordance with ethical principles for medical research involving humans (WMA, Declaration of Helsinki). The ethical research committee, the West IV *Comité de Protection des Personnes* (Nantes, France), approved the protocol version 1.0 of 26.12.2017 and its annexes 1 to 2, on February 13, 2018 (IdRCB n°2017- A03646-47; ClinicalTrials.gov Identifier: NCT03493243). All data were collected anonymously. After the receipt of appropriate information, all participants (and their legal guardians, for minors) freely signed a non-opposition form.

David V. Sheehan, as “copyright holder” of all versions of “*MINI International Neuropsychiatric Interview for Children and Adolescents*- M.I.N.I. Kid 7.0.2 (version 8/8/16)” authorized Nathalie Coulon, MD PhD at CHU Brest (France) and her team, as care providers, to use this interview under conditions that have been met.

### Statistical Analysis

For continuous variables, descriptive statistics included number of missing data, mean, standard deviation, median, quartile, minimum and maximum. For qualitative variables, frequency and percentage were used.

At the time of the study, one-year post-trauma, we compared participants with, vs. without, PTSD according to PCL-5 cut-off (primary endpoint), as well as teenagers who did, vs. did not, consult beyond the first week after the attack. The comparisons between participants were performed using comparison of means (Student test or Wilcoxon test) or frequencies (Chi-Square test or Fisher's exact test).

The concordance of the PTSD diagnosis was also sought by comparing the results of the PCL-5 and Mini-Kid via a calculation of Cohen's Kappa.

All analyses were carried out using SAS version 9.4 statistical software.

## Results

### Population

Table 1 shows the socio-demographic characteristics of the AVAL population, with the PCL-5 used to determine those with, vs. without PTSD. Of the target population (n = 53), 39 participants (73.6%) agreed to take part in this first observational step of what is expected to become the AVAL cohort study. The study population comprised 15 boys and 24 girls (sex ratio 1/1.6), with a median age of 16.9 years (16.0–18.0), all of French nationality, enrolled in a high school course at the time of the clinical evaluation.

**Table 1:** Socio-demographic characteristics of the AVAL Population.

**Characteristics**		**Total** ***N*** **=** **39**		**Without PTSD** ***N*** **=** **29**		**With PTSD** ***N*** **=** **10**		*P* ^*^
		***N***	**%**	***N***	**%**	***N***	**%**	
Gender	Male	15	38.5	14	48.3	1	10.0	0.0574
	Female	24	61.5	15	51.7	9	90	
	Sex Ratio	1/1.6						
Age	N collected	39	100	29	100	10	100	0.7371
Time of clinical evaluation	Mean ± SD	16.92 ± 0.41		16.92 ± 0.44		16.89 ± 0.31		
	Median (q1; q 3)	16.9 (16.6; 17.1)		16.8 (16.6; 17.1)		17.0 (16.8; 17.1)		
	Min; Max	16; 18		16; 18		16; 17		
Age	N collected	39	100	29	100	10	100	0.6833
Time of traumatic event	Mean ± SD	15.36 ± 0.54		15.34 ± 0.55		15.40 ± 0.52		
	Median (q1; q 3)	15.0 (15.0; 16.0)		15.0 (15.0; 16.0)		15.0 (15.0; 16.0)		
	Min; Max	15; 17		15; 17		15; 16		
Origin	France	39	100	29	100	10	100	
High School	First Year	2	5.1	1	3.4	1	10	0.4521
Time of clinical evaluation	Second Year	37	94.9	28	96.6	9	90	

*PTSD, Post-Traumatic Stress Disorder*.

* *Student Test or Mann-Whitney/Wilcoxon for quantitative variables*.

*Chi Squared Test or Fischer Exact Test for qualitative variables*.

Among the participants, 25.6% (*n* = 10) had a PCL-5 total score >32 and were therefore diagnosed with PTSD, 12 months after the attack. Socio-demographic characteristics (Table 1) were not statistically different between sample with (*n* = 10), vs. without (*n* = 29), PTSD.

### Clinical Impact

Table 2 shows the PCL-5 scores and their comparison for participants with, vs. without, PTSD. Those with PTSD had increased PCL-5 scores in total scale score (42.78 ± 8.12 vs. 14.11 ± 10.34, *p* < 0.0001) and the DSM5's four-factors: intrusion symptoms (12.10 ± 2.60 vs. 3.82 ± 3.58, *p* = 0.0001), avoidance symptoms (4.70 ± 2.31 vs. 1.93 ± 2.19, *p* = 0.0054), negative alterations in cognition and mood (12.10 ± 2.60 vs. 3.31 ± 3.62, *p* = 0.0003) and hyperarousal (13.90 ± 3.00 vs. 4.93 ± 3.44, *p* < 0.0001). PCL-5 provided an estimated PTSD prevalence of 25.6% (*n* = 10), whilst the PTSD prevalence was 28.2% (*n* = 11; 60 vs. 17.2%, *p* = 0.0167) with the MINI-kid. A moderate concordance of the diagnosis was observed between the two scales (κ 0.41 [0.09; 0.73]).

**Table 2:** Clinic of the AVAL population, Post traumatic symptomatology and comorbidity.

**Tools and questions, items**		**Total** ***N*** **=** **39**		**Without PTSD** ***N*** **=** **29**		**With PTSD** ***N*** **=** **10**		*P* ^*^
		***N***	**%**	***N***	**%**	***N***	**%**	
**PCL5**								
➢ Total score	N collected	37		28		9		<0.0001
	Mean ± SD	21.08 ± 15.82		14.11 ± 10.34		42.78 ± 8.12		
➢ Intrusion symptoms (Items 1–5)	N collected	38		28		10		0.0001
	Mean ± SD	6.00 ± 4.96		3.82 ± 3.58		12.10 ± 2.60		
➢ Avoidance symptoms (Items 6–7)	N collected	39		29		10		0.0054
	Mean ± SD	2.64 ± 2.51		1.93 ± 2.19		4.70 ± 2.31		
➢ Negative alterations in cognitions and mood (Items 8–14)	N collected	38		29		9		0.0003
	Mean ± SD	5.50 ± 5.49		3.31 ± 3.62		12.10 ± 2.60		
➢ Hyperarousal (Items 15–20)	N collected	39		29		10		<0.0001
	Mean ± SD	7.23 ± 5.16		4.93 ± 3.44		13.90 ±- 3.00		
**MINI kid 7.02**								
➢ Major depressive episode	Yes	15	38.5	8	27.6	7	70.0	0.0266
Past episode	Yes	14	93.3	7	87.5	7	100.0	1.000
➢ Suicidality	Yes	8	20.5	3	10.3	5	50.0	0.0164
Actual	Yes	7	87.5	2	66.7	5	100.0	0.3750
➢ Mood disorder with psychotic features, lifetime	Yes	4	10.3	1	3.4	3	30.0	0.0449
➢ Mood disorder with psychotic features, actual	Yes	2	5.1	0	0	2	20.0	0.0607
➢ Anxiety Disorder, at least one current disorder	Yes	20	51.3	12	41.4	8	80.0	0.0648
➢ Panic Disorder	Yes	6	15.4	1	3.4	5	50.0	0.0023
➢ Generalized Anxiety Disorder, current	Yes	2	5.1	0	0	2	20.0	0.0607
➢ Posttraumatic Stress Disorder	Yes	11	28.2	5	17.2	6	60.0	0.0167
➢ At least 1 current psychiatric disorder other than PTSD	Yes	25	64.1	15	51.7	10	100.0	0.0066
➢ Alcohol use disorder, past 12 months	No	38	97.4	28	96.6	10	100.0	1.0000
➢ Substance use disorder (no OH), past 12 months	No	37	94.9	28	96.6	9	100.0	0.4521
**Psychoactive substances use: close ended questions**								
➢ Change in cannabis consumption	Yes	3	7.7	1	3.4	2	20.0	0.0449
➢ Change in tobacco consumption	Yes	4	10.3	1	3.4	3	30.0	0.0284
➢ Change in alcohol consumption	Yes	16	41.0	12	41.4	4	40.0	0.8086

*PCL5, Post Traumatic Check List for DSM 5; MINI Kids, Mini International Neuropsychiatric Interview for Children and Adolescent; PTSD, Post Traumatic Stress Disorder*.

* *Student Test or Mann-Whitney/Wilcoxon for quantitative variables*.

*Chi Squared Test or Fischer Exact Test for qualitative variables*.

Table 2 also shows comparisons of those with, vs. without, PTSD across an array of psychiatric conditions, as determined by the MINI-kid. Although 74.4% (*n* = 29) of the participants did not meet PTSD criteria 1 year after the attack, 64.1% (*n* = 25) met criteria for a current, non-PTSD, psychiatric disorder. However, the comparison of with, vs. without, PTSD at one year post-attack showed those with PTSD to have a significantly increased prevalence of: (1) psychiatric comorbidities, namely major depressive episode (70 vs. 27.6%, *p* = 0.0266), suicidality (50 vs. 10.3%, *p* = 0.0164), mood disorder with psychotic features in lifetime (30 vs. 3.4%, *p* = 0.0449), panic disorder (50 vs. 3.4%, *p* = 0.0023); and (2) changes in the consumption of toxic substances, namely tobacco (30 vs. 3.4%, *p* = 0.0284) and cannabis (20 vs. 3.4%, *p* = 0.0449), but not alcohol.

### Impaired Functioning

Table 3 shows those with, vs. without, PTSD at 1 year post-attack to have impaired functioning across all measured domains, namely: school (significant impact in 50 vs. 13.8%; *p* = 0.0203), social life (significant impact in 40 vs. 0%, *p* < 0.0001) and family life (significant impact in 40 vs. 0%, *p* < 0.0001). Other indicators of impaired functioning in the PTSD group are also evident, including: (1) an increase in sensitivity to stressful events in the year after the attack (ALCES, hight stress in 70 vs 24.1%, *p* = 0.0409), (2) truancy for medical motives (44.4 vs 10.3%, *p* = 0.0407) and 3) a rise in the number of days with total incapacity of work in SDC (0.90 ± 1.29 vs. 0.10 ± 0.41, *p* = 0.0154), covering the week before assessment, and in Whodas 2.0 (4.50 ± 8.97 vs. 0.34 ± 1.86, *p* = 0.0070), covering the 30 days prior to assessment, or partial incapacity of work, both in SDC (2.63 ± 1.51 vs. 1.10 ± 2.09, *p* = 0.0110) and in Whodas 2.0 (18.90 ± 13.36 vs. 6.62 ± 11.78, *p* = 0.0134).

**Table 3:** School, social and family functioning in AVAL population.

**Tools and questions, items**			**Total** ***N*** **=** **39**		**Without PTSD** ***N*** **=** **29**		**With PTSD** ***N*** **=** **10**		*P* ^*^
			***N***	**%**	***N***	**%**	***N***	**%**	
**ALCES**									
➢ Total score since the attack	<150	Low Stress	13	33.3	12	41.1	1	10.0	0.0409
	Between 150 and 299	Moderate stress	12	30.8	10	34.5	2	20.0	
	>150	High Stress	14	35.9	7	24.1	7	70.0	
**Sheehan Disability Scale (SDC)**									
➢ Functional Impaiment in 3 major life domains									
1. School	0	No impact	12	30.8	12	41.4	0	0	0.0203
	1–3	Mild Impact	9	23.1	7	24.1	2	20.0	
	4–6	Moderate Impact	9	23.1	6	20.7	3	30.0	
	>7	Significant Impact	9	23.1	4	13.8	5	50.0	
2. Social life	0	No impact	18	46.2	18	62.1	0	0	<0.0001
	1–3	Mild Impact	11	28.2	9	31.0	2	20.0	
	4–6	Moderate Impact	6	15.4	2	6.9	4	40.0	
	>7	Significant Impact	4	10.3	0	0	4	40;0	
3. Family life	0	No impact	22	56.4	22	75.9	0	0	<0.0001
	1–3	Mild Impact	10	25.6	6	20.7	4	40.0	
	4–6	Moderate Impact	6	7.7	1	3.4	2	20.0	
	>7	Significant Impact	4	10.3	0	0	4	40.0	
➢ Days lost (in last week before the assessment)		Mean ± SD	0.31 ± 0.80		0.10 ± 0.41		0.90 ± 1.29		0.0154
➢ Days underproductive (in last week before the assessment)		Mean ± SD	1.43 ± 2.06		1.10 ± 2.09		2.63 ± 1.51		0.0110
**Whodas 2.0** (about past 30 days before the assessment)									
➢ Number of days with difficulties		Mean ± SD	13.64 ± 14.28		11.24 ± 14.15		20.60 ± 12.89		0.0921
➢ Days lost		Mean ± SD	1.41 ± 5.00		0.34 ± 1.86		4.50 ± 8.97		0.0070
➢ Days underproductive		Mean ± SD	9.77 ± 13.19		6.62 ± 11.78		18.90 ± 13.36		0.0134
**Question about truancy** (since the attack)		Yes	7	18.4	3	10.3	4	44.4	0.0407
**Treatment initiated since the attack**									
➢ Medical/psychological care in the first 7 days post-attack		Yes	38	97.4	28	96.6	10	100.0	1.0000
➢ Regular psychological or psychiatric follow up		Yes	14	35.9	7	24.1	7	70.0	0.0191
➢ Depression medication		Yes	2	5.1	0	0	2	20.0	0.0607
➢ Anxiety medication		Yes	6	15.4	3	10.3	3	30.0	0.1628
➢ Non drug therapy (relaxation/ EMDR/ hypnosis)		Yes	12	30.8	6	20.7	6	60.0	0.0427

*ALCES, Adolescent Life-Change Event Scale; WHODAS, World Health Organization Disability Assessment Scale*.

* *Student Test or Mann-Whitney/Wilcoxon for quantitative variables*.

*Chi Squared Test or Fischer Exact Test for qualitative variables*.

### Epidemiology

Table 4 shows factors significantly influencing PTSD development in this study. Pre-traumatic risk factors in those with, vs. without, PTSD were identified, including: (1) a previous history of trauma exposure, especially having witnessed a physical assault (60 vs 6.9%, *p* = 0.0014), or being concerned with a history of sexual assault (30 vs 3.4%, *p* = 0.0449); (2) personal or family history of mental disorder: personal history of depressive disorders (70 vs 27.6%, *p* = 0.0266), anxiety disorders (60 vs. 20.7%, *p* = 0.0427), psychiatric or psychological follow-up (80 vs. 37.9%, *p* = 0.0310); family history of: mental disorder (80 vs. 27.6%, *p* = 0.0073), depressive disorders (50 vs. 13.8%, *p* = 0.0151) or alcohol related disorders (40 vs. 6.9%, *p* = 0.0145). Post-traumatic risk factors in those adolescents who developed PTSD were personal, including psychiatric comorbidities, as shown in Table 2, and environmental such as a poor family support (30 vs 0%, *p* = 0.0131) or a poor school support (80 vs. 41.4%, *p* = 0.0211).

**Table 4:** Epidemiology, pre and post-traumatic risk factors, associated with development of PTSD.

**Tools and questions, items**		**Total** ***N*** **=** **39**		**Without PTSD** ***N*** **=** **29**		**With PTSD** ***N*** **=** **10**		*P* ^*^
		***N***	**%**	***N***	**%**	***N***	**%**	
**Life Event Checklist for DSM-5 (LEC-5)**								
➢ Physical assault (witnessed it)	Yes	8	20.5	2	6.9	6	60.0	0.0014
➢ Sexual assault (to be concerned with)	Yes	4	10.3	1	3.4	3	30.0	0.0449
**Genogram**								
➢ Personal history of mental disorder								
Depressive Disorders	Yes	15	38.5	8	27.6	7	70.0	0.0266
Anxiety Disorders	Yes	12	30.8	6	20.7	6	60.0	0.0427
Psychiatric or psychological follow up	Yes	19	48.7	11	37.9	8	80.0	0.0310
➢ Familial history of mental disorder								
All mental disorders combined	Yes	16	41.0	8	27.6	8	80.0	0.0073
Depressive disorders	Yes	9	23.1	4	13.8	5	50.0	0.0151
Alcohol related disorders	Yes	6	15.4	2	6.9	4	40.0	0.0145
**Family and School Resources** (since the attack)								
➢ Family support	No	3	7.7	0	0.0	3	30.0	0.0131
➢ School support	No	20	51.3	12	41.4	8	80.0	0.0211

*PTSD, Post Traumatic Stress Disorder*.

* *Student Test or Mann-Whitney/Wilcoxon for quantitative variables*.

*Chi Squared Test or Fischer Exact Test for qualitative variables*.

### Healthcare Pathways

Within 7 days following the attack (Table 3), 100% (*n* = 10) of adolescents who developed PTSD at one-year post-attack, received medical or psychological care through the medico-psychological emergency unit. Furthermore, at one one-year post-attack, 70% (*n* = 7) of the participants who met PTSD criteria, were also receiving current medical and/or psychological care (70 vs 24.1%, *p* = 0.0191), with 60% receiving non drug therapy, such as relaxation, *Eye Movement Desensitization and Reprocessing* (EMDR), and/or hypnosis (60 vs 20.7%, *p* = 0.0427).

Table 5 summarizes the comparison between teenagers who did (*n* = 20), vs. did not (*n* = 19), consult beyond 1 week after the attack. Only the prevalence of impaired concentration (80 vs. 47.4%, *p* = 0.0337), days of reduced effectiveness (2.21 ± 2.25 vs. 0.61± 1.50, *p* = 0.0161) and personal history of anxiety disorders (65 vs. 31.6%, *p* = 0.0369), were more prevalent in the followed up sample, with no statistically significant differences evident regarding gender (female 65 vs. 57.9%, *p* = 0.6485), prior history of traumatic exposure (65 vs. 36.8%, *p* = 0.0787), prior history of depressive disorders (50 vs. 26.3%, *p* = 0.1286) or self-perceived significant impact of the attack on their education (30 vs. 15.8%, *p* = 0.4506), their social life (20 vs 0%, *p* = 0.1060) or their family life (20 vs 0%, *p* = 0.1060).

**Table 5:** Care for teenagers who did or did not consult beyond 1 week, after the attack.

**Tools and questions, items**			**Total** ***N*** **=** **39**		**Without PTSD** ***N*** **=** **29**		**With PTSD** ***N*** **=** **10**		*P* ^*^
			***N***	**%**	***N***	**%**	***N***	**%**	
**Gender**									
➢ Male		Yes	15	38.5	8	42.1	7	35.0	0.6485
➢ Female		Yes	24	61.5	11	57.9	13	65.0	
**Genogram**									
➢ **Personal history of mental disorder**									
Depressive Disorder		Yes	15	38.5	5	26.3	10	50.0	0.1286
Anxiety Disorders		Yes	19	48.7	6	31.6	13	65.0	0.0369
Psychiatric or psychological follow up		Yes	12	30.8	6	31.6	6	30.0	0.9150
**Life Event Checklist for DSM-5 (LEC-5)**									
➢ Victim of a stressful situation before the attack		Yes	20	51.3	7	36.8	13	65.0	0.0787
➢ Witness a stressful situation before the attack		Yes	17	43.6	8	42.1	9	45.0	0.8554
**Sheehan Disability Scale (SDC)**									
➢ Functional Impaiment in 3 major life domains									
1. School	<7	Moderate Impact	30	76.9	16	84.2	14	70.0	0.4506
	≥7	Significant Impact	9	23.1	3	15.8	6	30.0	
2. Social life	<7	Moderate Impact	35	89.7	19	100.0	16	80.0	0.1060
	≥7	Significant Impact	4	10.3	0	0.0	4	20.0	
3. Family life	<7	Moderate Impact	35	89.7	19	100.0	16	80.0	0.1060
	≥7	Significant Impact	4	10.3	0	0.0	4	20.0	
➢ Days lost (in last week befre the assessment)		Mean ± SD	0.31 ± 0.80		0.05 ± 0.23		0.55 ± 1.05		0.0513
➢ Days underproductive (in last week befre the assessment)		Mean ± SD	1.43 ± 2.06		0.61 ± 1.50		2.21 ± 2.25		0.0161
**Clinic**	Impaired concentration	Yes	25	64.1	9	47.4	16	80.0	0.0337

* *Student Test or Mann-Whitney/Wilcoxon for quantitative variables*.

*Chi Squared Test or Fischer Exact Test for qualitative variables*.

## Discussion

### Main Findings

Twelve months after being directly exposed to the terrorist attack perpetrated in London on March 22^nd^, 2017, 25.6% of the teenagers in our sample met DSM-5 criteria for PTSD. Concurrent to PTSD, elevations in psychiatric comorbidities, changes in consumption of toxic substances, and impaired functioning in all areas of their life were also evident. Other than PTSD, 64.1% of the participants also expressed a current psychiatric disorder. Apart from an increased prevalence of impaired concentration, days of reduced effectiveness and personal history of anxiety disorders, in the sample which had received mental health care, there were no statistically significant differences did, vs. did not, consult beyond 1 week after the attack.

Notably, few studies have investigated a similar target population (adolescents aged 16 years and directly exposed to a terrorist attack) or with the same experimental protocol (intentional and man-made event; clinical assessment at 1 year). The PTSD prevalence of 25.6% in our study (with 25.6% satisfying criteria for intrusive, avoidance and hyperarousal symptoms and 23.1% satisfying criteria for negative alterations in cognition and mood) is consistent with the overall literature in this area, including in children, adolescents (4) and adults (6, 7). A large sample of Norwegian high school (27) students were assessed following the Oslo terror attack on July 22^nd^, 2011 (*n* = 10,220; mean age 16.9 years; 53% of girls), although only 1.8% of this sample were on site. Consequently, PTSD prevalence varied according to exposure level, with only 0.8% of all respondents reporting substantial distress on the reexperiencing item (DSM IV), 4.9% on the avoidance item, 1.1% on the hyperarousal item and 0.4% on all three areas, approximately 7 months after the event. Following the World Trade Center Attack, a sample of 817 adolescents, aged 13–18 years, was drawn from a Jewish high school, although these young people were not directly exposed to the attack. Consequently, at 1 year after the attack, only 2.3% seemed to meet PTSD criteria (28). Among adults involved in the terror attacks in January 2015 in Paris (civilian, *n* = 190, median age 41), 31% of directly threatened participants met PTSD criteria at 6 months (29).

Other psychological disorders were also evident at 1 year in our target population, which is also in accordance with the literature (2, 3, 8, 9). A systematic review (30) suggested the risk of major depressive episode, in adults directly exposed to a terrorist attack, ranged from 20 to 30% in the first months after its occurrence. Chemtob et al. (31) also found an association between exposure to terrorism and PTSD, suicidal ideation and functional impairment in a sample of 2,094 Israeli adolescents, aged 12 to 18 years. At 1 year following the World Trade Center attack, adolescents with PTSD showed an increased risk for suicidal ideation (28) and after 18 months, increased substance use was associated with impaired school performance (11). In the Paris, 2015 attacks, within the 58 directly threatened civilians (29), 19% had depression, 38% anxiety disorder and 19% increased suicide risk, at 6 months after the attacks. Given the high prevalence of 38.5% of participants with PTSD having a depressive episode and 20.5% having suicidal ideation in our target population, it may be important to note that data collection occurred around the first anniversary date on the event. Consequently, data collection occurred at an emotionally heightened time-point, although the attacks in London were more discreetly commemorated in France, in contrast to the widely commemorated Paris, 2015 attacks.

Epidemiologically, the risk factors for PTSD in our population were generally in agreement with the wider literature. In a meta-analysis, Brewin et al. (32) classified risk factors according to the strength of their predictive effects in three categories. (1) Some factors, such as gender, did not appear in all studies, although a consistent pattern for women to be at higher risk for PTSD than men was evident. In our study, both groups (with and without PTSD), included more girls but the groups did not statistically differ on gender. (2) Other factors, such as previous trauma, predicted PTSD more consistently, with variations at least partly being explained by the methodology used. In our work, having witnessed a physical assault was statistically significant in predicting the development of PTSD. (3) Factors such as personal or family psychiatric history, childhood abuse, had a more uniform predictive effect, and the significant effects of these factors were also present in our results. In our study, depressive disorders were again noted here, as a risk factor for PTSD such as personal or familial history of mental disorders.

However, several studies have enhanced the stronger influence of peri- and post-traumatic factors, than pre-traumatic factors (13, 32–34), on PTSD risk. Trickey et al. (13) added the impact of superior subjective experience to the objective elements, even if as in the situation studied, strong objective elements were present such as the severity of the events and the level of exposure (the car just stopped a few meters away from the participants). As to the importance of post-trauma factors, it is notable that in our study, low social support was significantly associated with PTSD risk at 1 year. This is similar to the meta-analysis by Trickey et al. (13), which focussed on children and adolescents, as well as with data on the first responders at 12 months after Paris terror attacks (34) in November 2015, or in a systematic review (35) about consequences of 9/11 on children's and young adult's mental health. About adolescents, in this last review of Rousseau et al. (35), low family support as parental unavailability to discuss the events or parent-adolescent conflict was highlighted. By way of qualifying social support as a predictor, in addition, with a comparison of two meta-analysis (32, 33), social support appeared a stronger predictor when the event had happened at least 3 years before the moment of the study.

According to our results, it is also worth mentioning these PTSD risk factors did not overlap with the effects of predictive factors as to whether follow up did, vs. did not, occur beyond 1 week.

As a consequence of France's experience of wars, disasters, and terrorist attacks, medico-psychological units have been deployed since July 1995, providing initial and longer-term support (36, 37). Such immediate and longer-term support was provided for the attacks in Paris 2015 (29, 36) and in Nice 2016, where more than 30,000 people were present, including babies, children and adolescents (38, 39). The Nice attack, with a young population impacted (39) (in the early care phase, 365 children and adolescents were registered at the pediatric medico-psychological emergency unit with 25.5% <6 years old, 44.7% 6–11, and 29.8% 12–17 years old) and experience of Lenval University Children's Hospital, is relevant to our work. It was indeed observed a great number of families (39) spontaneously converged to Children's Hospital in search of psychological or medical help, as in our work where all teenagers with PTSD 1 year after the attack received post-immediate care and 70% were still being followed up at the time of our study. The Nice study (39), like ours, highlighted and encouraged the follow-up of adolescents, especially if they are vulnerable, in a standardized manner, to ensure the screening and treatment of all young people.

Cohort studies conducted following other terrorist attacks, such as 9/11, the Madrid, 2004 car bombings, the 2011 Oslo/Utoya massacre and Paris, 2015, showed participation rates ranging from 40 to 70% in the first wave, and 50 to 75% in the second wave (40, 41). Although these attacks vary considerably in their details, the participation rates are similar, and may be improved in younger people via the utilization of social media, peers, and educational environments, as noted by Vuillermoz et al. following the Paris, 2015 attacks (41). The higher level (73.6 %) of participation in the current study could be explained by various details: (1) the small size of the total target population which allowed us to identify each teenager present in the event; (2) links with the network set up at the local level (high-school, academy and diocese, partners of justice, medico-psychological emergency unit, care, and political bodies); (3) awareness of all the partners in order to deploy a prevention and care message, when the research was launched, in an information meeting in the high school; (4) personalized phone call if necessary; and (5) creation of a specific email address, allowing participants to engage in discussions, including with family and high-school staff. Such a simple provision may be especially important for an adolescent population. Moreover, beyond the research dimension, raising awareness in the network about adolescents after the attack, involving parents, professors, but also political actors, seemed essential to us to build this social link, which is recognized as positive in the prognosis (13, 34, 35). Our idea was already to open the discussion so that young people in difficulty do not remain isolated. On several occasions, contact details of professionals to support these teenagers were sent and some were then able to benefit from an adapted and specialized follow up. Regarding adolescent psychopathology, in fact, taking the first step is sometimes necessary to initiate care.

Furthermore, about care and therapeutics, a whole new literature is appearing: for example, current research focuses on the treatment with ketamine of major depressive episodes, suicidal ideation (42–44) and chronic PTSD (45, 46) in adult populations. Common pathophysiological factors could indeed be involved in the complexity of PTSD, but also in the depressive episode, suicidality (42). In France in addition, suicide is the second leading cause of death for young people aged 15 to 24 (47). Nevertheless, ketamine is beginning to be explored in pediatrics in acute pain (48), but studies will be continued and questioned for PTSD and suicidality in children, teenagers. The therapeutic work also remains non negligible and Bianchini et al. (49) showed a significantly decreased in PTSD symptomatology and psychological distress severity with Cognitive Behavior Therapy in 39 young subjects, followed and evaluated 1 year after l'Aquila Earthquake.

### Strengths and Limitations

One of the great strengths of the study was the scarcity of the population studied within the literature (teenagers directly exposed to a terrorist attack) and the search for exhaustiveness in the recruitment of participants. To our knowledge, only one cohort in Europe has been conducted to assess impact of a terrorist attack on a directly exposed adolescent population (2011 Norway attacks) and the rate participation of our study was one of the best, due to a precise configuration, detailed previously. Another strength of our work was the protocol built with self-administered and clinician-administered questionnaires, derived from the literature and previous cohorts following terrorist attacks. A few tools (LEC-5, ALCES, Sheehan Disability Scale, Social Provisions Scale-Short Form, PCL-5, MINI-kid 7.0.2, WHODAS 2.0) were structured and validated, allowing for validated international comparisons across traumas. Moreover, to limit biases, all clinician-administered questionnaires were completed by a single investigator, with the supervision of two senior doctors. The protocol was specifically built, in a short time, to give to the target population messages of prevention and to orient, if necessary, toward therapeutic spaces, whilst also allowing for systematic investigation to occur.

Despite these strengths, several limitations should be noted. First, the entire target population was comprised of only 53 students, this small sample size being a clear limitation, although the participation rate was remarkable. Second, the study took place 1 year after the attack, due to the time required to create the protocol and obtain its authorization. As data collection ended up matching with the first anniversary of the attack, symptoms of PTSD, depression and anxiety may have been exacerbated. Third, the study design was cross-sectional, observational and descriptive. The associations of PTSD and each pre-, peri- and post-traumatic factor were not derived via a multivariate model. Consequently, the results did not include estimates of odds ratio (OR) and their 95% confidence interval limits. Fourth, social support was an important protective factor for PTSD development, and the study only gathered the testimony of study participants at one moment in time. It would be interesting to complete this observation on the on a longitudinal model to assess the teenagers' evolution. To supplement by the observation of the subjects who were indirectly exposed to the trauma (family, relatives, friends, etc…) could at last increase knowledge about trauma in adolescents.

### Implications

This work has the particularity of studying an extremely specific population, namely adolescents directly exposed to a terrorist attack. The study provides a protocol for the integration of rapidly responding care with systematic research that builds on previous literature, whilst providing pointers for improvements in the mental healthcare of this adolescent population. The emergence of other psychiatric conditions in those with PTSD at one-year post-trauma, including mood disorders and substance use/abuse, highlights the importance of early intervention. Future research will contribute to improve such intervention.

## Data Availability Statement

The raw data supporting the conclusions of this article will be made available by the authors, without undue reservation.

## Ethics Statement

The studies involving human participants were reviewed and approved by Comité de Protection des Personnes Ouest IV-Nantes. Written informed consent to participate in this study was provided by the participants' legal guardian/next of kin.

## Author Contributions

MG, J-PS, and NC co-conceived the study. MG designed the protocol of the study with the methodological and scientific support of NC. J-PS proofread the manuscript. MC planned the statistical analysis in agreement with NC. MC performed all the statistical analyses. NC synthesized all the results and draft the manuscript. All authors contributed to the evolution of the manuscript and approved the final version.

## Funding

For the investigator and volunteers from the departmental medico-psychological emergency unit, travel, accommodation and catering were respectively covered by the military hospital *Clermont-Tonnerre* (Brest, France) and the University Civil Hospital of Brest. An association for research in Psychiatry (ABREP *Association Brestoise pour la Recherche en Psychiatrie*) also contributed to data collection costs.

## Conflict of Interest

The authors declare that the research was conducted in the absence of any commercial or financial relationships that could be construed as a potential conflict of interest.

## Publisher's Note

All claims expressed in this article are solely those of the authors and do not necessarily represent those of their affiliated organizations, or those of the publisher, the editors and the reviewers. Any product that may be evaluated in this article, or claim that may be made by its manufacturer, is not guaranteed or endorsed by the publisher.

## References

[1] Bui E Ohye B Palitz S Olliac B Goutaudier N Raynaud JP. Acute and chronic reactions to trauma in children and adolescents. In IACAPAP (International Association for Child and Adolescents Psychiatry and Allied Professions) Textbook, Anxiety Disorders, Chapter F.4. Geneva (2014).

[2] Fremont WP. Childhood reactions to terrorism-induced trauma: a review of the past 10 years. J Am Acad Child Adolesc Psychiatry. (2004) 43:381–92. 10.1097/00004583-200404000-00004 15187798

[3] Saraiya A Garakani A Billick SB. Mental health approaches to child victims of acts of terrorism. Psychiatr Q. (2013) 84:115–24. 10.1007/s11126-012-9232-4 22736303

[4] Wang CW Chan CLW Ho RTH. Prevalence and trajectory of psychopathology among child and adolescent survivors of disasters: a systematic review of epidemiological studies across 1987-2011. Soc Psychiatry Psychiatr Epidemiol. (2013) 48:1697–720. 10.1007/s00127-013-0731-x 23824234

[5] APA, American Psychiatric Association. Diagnostic and Statistical Manual of Mental Disorders (DSM IV-R). Fourth Edition Revised. American Psychiatric Association, Washington, D.C. (2000).

[6] Santiago PN Ursano RJ Gray CL Pynoos RS Spiegel D Lewis-Fernandez R. A Systematic Review of PTSD Prevalence and Trajectories in DSM-5 Defined Trauma Exposed Populations:Intentional and Non-Intentional Traumatic Events. PLoS ONE. (2013) 8:59236. 10.1371/journal.pone.0059236 23593134 PMC3623968

[7] Carlson F Van Ommeren M Flaxman A Cornett J Whiteford H Saxena S. New WHO prevalence estimates of mental disorders in conflict settings : a systematic review and meta-analysis. Lancet. (2019) 394:240–48. 10.1016/S0140-6736(19)30934-1 31200992 PMC6657025

[8] Pfefferbaum B Stuber J Galea S Fairbrother G. Panic reactions to terrorist attacks and probable posttraumatic stress disorder in adolescents. J Trauma Stress. (2006) 19:217–28. 10.1002/jts.20118 16612814

[9] Comer JS Kendall PC. Terrorism. The psychological impact on youth. Clin Psychol Sci Pract. (2007) 14:179–212. 10.1111/j.1468-2850.2007.00078.x

[10] Schiff M Zweig HH Benbenishty R Hasin DS. Exposure to terrorism and Israeli youths' cigarette, alcohol, and cannabis use. Am J Public Health. (2007) 97:1852–8. 10.2105/AJPH.2006.090514 17761574 PMC1994181

[11] Chemtob CM Nomura Y Josephson L Adams RE Sederer L. Substance use and functional impairment among adolescents directly exposed to the 2001 World Trade Center attacks. Disasters. (2009) 33:337–52. 10.1111/j.1467-7717.2008.01077.x 19178553

[12] Pollice R Bianchini V Roncone R Casacchia M. Marked increase in substance use among young people after l'Aquila earthquake. Eur Child Adolesc Psychiatry. (2011) 20:429–30. 10.1007/s00787-011-0192-2 21674248

[13] Trickey D Siddaway AP Meiser-Stedman R Serpell L Field AP. A meta-analysis of risk factors for post-traumatic stress disorder in children and adolescents. Clin Psychol Rev. (2012) 32:122–38. 10.1016/j.cpr.2011.12.001 22245560

[14] Grenon M Consigny M Lemey C Simson JP Coulon N. Impact of a terrorist attack on the mental health of directly exposed French adolescents: study protocol for the first step of the AVAL Cohort Study. Front Psychiatry. (2019) 10:744. 10.3389/fpsyt.2019.00744 31708812 PMC6823664

[15] American Psychiatric Association. Diagnostic and Statistical Manual of Mental Disorders (DSM 5). Fifth Edition, American Psychiatric Association, Washington, D.C. (2013).

[16] Gray MJ Litz B Hsu JL Lombardo TW. Psychometric properties on the life events checklist. Assessment. (2004) 11:330–41. 10.1177/1073191104269954 15486169

[17] Yeaworth RC McNamee MJ Pozehl B. The adolescent life change event scale: its development and use. Adolescence. (1992) 27:783–802. 1471559

[18] Sheehan KH Sheehan DV. Assessing treatment effects in clinical trials with the discan metric of the Sheehan Disability Scale. Int Clin Psychopharmacol. (2008) 23:70–83. 10.1097/YIC.0b013e3282f2b4d6 18301121

[19] Leon AC Olfson M Portera L Farber L Sheehan DV. Assessing psychiatric impairment in primary care with the Sheehan Disability Scale. Int J Psychiatry Med. (1997) 27:93–105. 10.2190/T8EM-C8YH-373N-1UWD 9565717

[20] Caron J. Une validation de la forme abrégée de l'Échelle de provisions sociales : l'EPS-10 items. Sante Ment Que. (2013) 38:297–318. 10.7202/1019198ar 24337002 PMC5031489

[21] Ashbaugh AR Houle-Johnson S Herbert C El-Hage W Brunet A. Psychometric Validation of the English and French Versions of the Posttraumatic Stress Disorder Checklist for DSM-5 (PCL-5). Plos ONE. (2016) 11: e0161645. 10.1371/journal.pone.0161645 27723815 PMC5056703

[22] Brunet A Weiss DS Metzler TJ Best SR Neylan TC Rogers C. et al. The Peritraumatic Distress Inventory: a proposed measure of PTSD criterion A2S. Am J Psychiatry. (2001) 158:1480–5. 10.1176/appi.ajp.158.9.1480 11532735

[23] Weathers FW Litz BT Herman DS Huska JA Keane TM. The PTSD checklist: reliability, validity, and diagnostic utility, IX^th^ Annual Meeting of the International Society for Traumatic Stress Studies, San Antonio, TX (1993).

[24] McDonald SC Calhoum PS. The diagnostic accuracy of the PTSD checklist: a critical review. Clin Psychol Rev. (2010) 30:976–87. 10.1016/j.cpr.2010.06.012 20705376

[25] Blevins CA Weathers FW Davis MT Witte TK Domino JL. The Posttraumatic Stress Disorder Cheklist for DSM-5 (PCL-5): Development and Initial Psychometric Evaluation. J Trauma Stress. (2015) 28:489–98. 10.1002/jts.22059 26606250

[26] Sheehan DV Lecrubier Y Sheehan KH Amorim P Janavs J Weiller E. The Mini-International Neuropsychiatric Interview (M.I.N.I.): the development and validation of a structured diagnostic psychiatric interview for DSM-IV and ICD-10. J Clin Psychiatry. (1998) 59 Suppl 20: 22–33; quiz 34-57. 9881538

[27] Nordanger DØ Hysing M Posserud MB Lundervold AJ Jakobsen R Olff M. Posttraumatic responses to the July 22, 2011 Oslo Terror among Norwegian high school students. J Trauma Stress. (2013) 26:679–85. 10.1002/jts.21856 24243587

[28] Chemtob CM Madan A Berger P Abramovitz R. Adolescent exposure to the world trade center attacks, PTSD symptomatology, and suicidal ideation. J Trauma Stress. (2011) 24:526–9. 10.1002/jts.20670 21882245

[29] Vandentorren S Pirard P Sanna A Aubert L Motreff Y Dantchev N. Healthcare provision and the psychological, somatic and social impact on people involved in the terror attacks in january 2015 in Paris: Cohort Study. Br J Psychiatry. (2018) 212:207–14. 10.1192/bjp.2017.63 29557760

[30] Salguero JM Fernandez-Berrocal P Iruarrizaga I Cano-Vindel A Galea S. Major depressive disorder following terrorist attacks: a systematic review of prevalence, course and correlates. BMC Psychiatry. (2011) 11:96. 10.1186/1471-244X-11-96 21627850 PMC3120744

[31] Chemtob CM Pat-Horenczyk R Madan A Pitman SR Wang Y Doppelt O. Israeli adolescents with ongoing exposure to terrorism: suicidal ideation, posttraumatic stress disorder, and functional impairment. J Trauma Stress. (2011) 24:756–9. 10.1002/jts.20708 22162099

[32] Brewin CR Andrews B Valentine JD. Meta-analysis of risk factors for posttraumatic stress disorder in trauma-exposed adults. J Consult Clin Psychol. (2000) 68:748–66. 10.1037/0022-006X.68.5.748 11068961

[33] Ozer EJ Best SR Lipsey TL Weiss DS. Predictors of posttraumatic stress disorder and symptoms in adults: a meta-analysis. Psychol Bull. (2003) 129:52–73. 10.1037/0033-2909.129.1.52 12555794

[34] Motreff Y Baubet T Pirard P Rabet G Petitclerc M Eilin Stene L. Factors Associated with PTSD and Partial PTSD Among First Responders Following the Paris Terror Attacks in November 2015. J Psychiatr Res. (2020) 121:143–50. 10.1016/j.jpsychires.2019.11.018 31821960

[35] Rousseau C Jamil U Bhui K Boudjarame M. Consequences of 9/11 and the war onterror on children and young adult's mental health: a systematic review of the past 10 years. Clin Child Psychol Psychiatry. (2015) 20:173–93. 10.1177/1359104513503354 24068751

[36] Prieto N Cheucle E Faure P Digard F Dalphin C Pachiaudi V. Defusing of victims of the terrorist attacks in paris. Elements of assessment one-month post-event. Encephale. (2018) 44:118–21. 10.1016/j.encep.2016.10.002 28041691

[37] Verger P Dab W Lamping DL Loze JY Deschaseaux-Voinet C Abenhaim L. The Psychological Impact of Terrorism: An Epidemiologic Study of Posttraumatic Stress Disorder and Associated Factors in Victims of the 1995-1996 Bombings in France. Am J Psychiatry. (2004) 161:1384–9. 10.1176/appi.ajp.161.8.1384 15285963

[38] Chauvelin L Gindt M Olliac B Robert P Thümmler S Askenasy F. Emergency Organization of child psychiatric care following the terrorist attack on July 14, 2016, in Nice, France. Disaster Med Public Health Prep. (2019) 13:144–6. 10.1017/dmp.2018.51 29916338

[39] Askenazy F Gindt M Chauvelin L Battista M Guenolé F Thümmler S. Early Phase psychiatric response for children and adolescents after mass trauma: lessons learned from the truck-ramming attack in Nice on July 14^th^ 2016. Front Psychiatry. (2019) 10: 65. eCollection 2019. 10.3389/fpsyt.2019.00065 30873048 PMC6401610

[40] Durodié B Wainwright D. Terrorism and Post-Traumatic Stress Disorder: A Historical Review. Lancet Psychiatry. (2019) 6:61–71. 10.1016/S2215-0366(18)30335-3 30342864 PMC9939936

[41] Vuillermoz C Eilin Stene L Aubert L Motreff Y Pirard P Baubet T. Non-participation and attrition in a longitudinal study of civilians exposed to the January 2015 terrorist attacks in Paris, France. BMC Med Res Methodol. (2020) 20:63. 10.1186/s12874-020-00943-x 32171236 PMC7071581

[42] De Berardis D Fornaro M Valchera A Cavuto M Perna G Di Nicola M. Eradicating Suicide at Its Roots: Preclinical Bases and Clinical Evidence of the Efficacy of Ketamine in the Treatment of Suicidal Behaviors. Int J Mol Sci. (2018) 19:2888. 10.3390/ijms19102888 30249029 PMC6213585

[43] Orsolini L Latini R Pompili M Serafini G Volpe U Vellante F. Understanding the Complex of Suicide in Depression: from Research to Clinics. Psychiatry Investig. (2020) 17:207–21. 10.30773/pi.2019.0171 32209966 PMC7113180

[44] Fu DJ Ionescu DF Li X Lane R Limp P Sancora G. Esketamine nasal spray for rapid reduction of major depressive disorder symptoms in patients who have active suicidal ideation with intent:double-blind, randomized study (Aspire I). J Clin Psychiatry. (2020) 81:19m13191. 10.4088/JCP.19m13191 32412700

[45] Feder A Rutter SB Schiller D Charney DS. The emergence of ketamine as a novel treatment for posttraumatic stress disorder. Adv Pharmacol. (2020) 89:261–86. 10.1016/bs.apha.2020.05.004 32616209

[46] Feder A Costi S Rutter SB Govindarajulu U Jha MK Horn SR. A randomised controlled trial of repeated ketamine administration for chronic posttraumatic stress disorder. Am J Psychiatry. (2021) 178:193–202. 10.1176/appi.ajp.2020.20050596 33397139

[47] Haute Autorité de Santé. Recommander les bonnes pratiques. Notes de cadrage. Tentatives de suicide et risque suicidaire chez l'enfant et l'adolescent : prévention, évaluation, prise en charge. (2020). Available online at: https://www.has-sante.fr/upload/docs/application/pdf/2020-07/reco348_cadrage_tentative_suicide.pdf (accessed January 22, 2022).

[48] Silva LO Lee JY Bellolio F Homme JL Anderson JL. Intranasal ketamine for acute pain management in children: a systematic review and meta-analysis. Am J Emerg Med. (2020) 38:1860–1866. 10.1016/j.ajem.2020.05.094 32739857 PMC7572639

[49] Bianchini V Roncone R Tomassini A Necozione S Cifone MG Casacchia M. Cognitive behavioral therapy for young people after l'Aquila earthquake. Clin Pract Epidemiol Ment Health. (2013) 9:238–42. eCollection 2013. 10.2174/1745017901309010238 24358053 PMC3866707

